# Cloud Servers: Resource Optimization Using Different Energy Saving Techniques

**DOI:** 10.3390/s22218384

**Published:** 2022-11-01

**Authors:** Mohammad Hijji, Bilal Ahmad, Gulzar Alam, Ahmed Alwakeel, Mohammed Alwakeel, Lubna Abdulaziz Alharbi, Ahd Aljarf, Muhammad Umair Khan

**Affiliations:** 1 Faculty of Computers & Information Technology, University of Tabuk, Tabuk 71491, Saudi Arabia; 2 Warwick Manufacturing Group, University of Warwick, Coventry CV4 7AL, UK; 3 School of Computing, Ulster University, Belfast BT15 1AP, UK; 4 College of Computers & Information Systems, Umm Al Qura University, Mecca 21955, Saudi Arabia; 5 School of Computing, Gachon University, Seongnam-si 13120, Korea

**Keywords:** cloud computing, distributed systems, data centers, virtual machines, energy saving

## Abstract

Currently, researchers are working to contribute to the emerging fields of cloud computing, edge computing, and distributed systems. The major area of interest is to examine and understand their performance. The major globally leading companies, such as Google, Amazon, ONLIVE, Giaki, and eBay, are truly concerned about the impact of energy consumption. These cloud computing companies use huge data centers, consisting of virtual computers that are positioned worldwide and necessitate exceptionally high-power costs to preserve. The increased requirement for energy consumption in IT firms has posed many challenges for cloud computing companies pertinent to power expenses. Energy utilization is reliant upon numerous aspects, for example, the service level agreement, techniques for choosing the virtual machine, the applied optimization strategies and policies, and kinds of workload. The present paper tries to provide an answer to challenges related to energy-saving through the assistance of both dynamic voltage and frequency scaling techniques for gaming data centers. Also, to evaluate both the dynamic voltage and frequency scaling techniques compared to non-power-aware and static threshold detection techniques. The findings will facilitate service suppliers in how to encounter the quality of service and experience limitations by fulfilling the service level agreements. For this purpose, the CloudSim platform is applied for the application of a situation in which game traces are employed as a workload for analyzing the procedure. The findings evidenced that an assortment of good quality techniques can benefit gaming servers to conserve energy expenditures and sustain the best quality of service for consumers located universally. The originality of this research presents a prospect to examine which procedure performs good (for example, dynamic, static, or non-power aware). The findings validate that less energy is utilized by applying a dynamic voltage and frequency method along with fewer service level agreement violations, and better quality of service and experience, in contrast with static threshold consolidation or non-power aware technique.

## 1. Introduction

This Virtualization techniques distribute the physical server into many remote and single-performance computer system environments by implementing a layer like a hypervisor or virtual machine manager on hardware or operating systems. In the implemented performance environment, every single-performance computer, such as a virtual machine, runs freely, combined with an operating system and other relevant applications devoid of mutual interference. The virtualization method was not trendy before due to some challenges, such as separate hardware resources, memory, and inadequate network [1,2,3].

Virtualization has emerged with advancements in technology, such as enhancements in hardware, cloud computing, IT networks, etc. [4,5]. The research community and practitioners started to work on the effective operation of virtualization when more users’ demands and use of cloud data centers for performing their tasks with other applications increased [6,7]. Issues were raised, such as overloaded and idle servers; if one server fails to operate, then all virtual machines will be affected, protection of virtual machines and hardware failure, etc. These issues were resolved with the beginning of virtual machine migration initiated from process migration [8]. The greater part of cloud computing operations is encouraged by virtual machine migration, such as server consolidation, hardware maintenance, energy, and flow management [9,10,11].

Numerous cloud computing models have been developed in which control and management of computing resources are provided. This helps businesses and clients use resources according to their design needs [12,13,14]. As an alternative to acquiring increased amounts in obtaining information technology infrastructure and dealing with hardware and software maintenance and updates, companies can outsource their computational requirements to the cloud. Large-size data centers have developed that consist of thousands of processing nodes and expend massive volumes of electric power. According to the latest survey, information technology impacts 25% of the total cost of managing and using data centers [15,16].

Energy consumption is overwhelming not only due to idle computing resources but also because of the ineffective management of these computational hardware and software resources. Servers commonly operate up to 50% complete capacity ahead of additional costs on over-provision and total cost of acquisition [17]. Energy management can be used to leverage resources through virtualization techniques and technology [18,19]. It permits cloud providers to generate many virtual machine occurrences on a separate physical server to enhance the efficient management and utilization of computational resources. This will increase the return on investment.

Amiri et al. [20] recommended an SDN (Software Defined Network) model for choosing DC (Data Centers) for novel gaming sessions. They used a hierarchy-based model for transport/response delay with bandwidth status by using the Lagrange algorithm and logarithmic techniques. Similarly, they used a new approach to reduce end-to-end latency in a cloud-based gaming data center environment [21]. Cai et al. [22] conducted a comprehensive survey on cloud gaming by involving various facets such as the platform used for cloud gaming, various optimization techniques, and commercial services for cloud gaming. Further, they explored the experience factor for gamers and energy utilization with network metrics. Chen et al. [23] proposed an approach for describing energy usage for virtual machines using measurement attributes such as performance, execution time, power (utilization and effectiveness), and energy usage. Therefore, to reduce the cost related to the cloud and to improve energy saving needed appropriate optimization techniques to enhance the user gamer experience.

*GreenCloud* architecture aims to reduce data center power consumption while guaranteeing performance from the users’ perspective. *GreenCloud* architecture enables comprehensive online monitoring, live virtual machine migration, and VM placement optimization. For experimentation, the CloudSim framework is used. CloudSim is a free and open-source framework based on Java language used for cloud computing infrastructure and services simulations. Similarly, this framework is utilized to model and simulate a cloud computing setting to perform tests and produce results. Further, it maintains various functionalities such as the generation of cloud-based entities, relations among entities, processing events, jobs and tasks queue, and implementation of broker policies [24,25].

The major contribution of the proposed research will be as follows:

- To investigate how resource optimization can be performed in gaming data centers
- Utilizing real-time gaming workload
- To measure service quality during online gaming data by utilizing its two features, i.e., energy consumption and SLA (Service Level Agreement)
- To test and implement DVFS (Dynamic Voltage and Frequency Scaling), non-power aware, and static threshold virtual machine consolidation techniques for improving service quality.

The remainder of the paper is organized as follows: Section 2 explains the literature review, followed by Section 3.1, which presents challenges related to the migration of a single virtual machine; Section 3.2 addresses the challenges related to the migration of the dynamic virtual machine; Section 4 discusses system methodology; Section 5 describes performance analysis and discussion while Section 6 represents conclusions and future work close the article.

## 2. Literature Review

Nathuji and Schwan [26] did initial work on the application of power management in virtualized data centers by proposing an architecture called a data center resource management system by splitting it into two categories: local policies and global policies. Then, [27] worked on virtualized heterogeneous environment power management and proposed the problem of sequential optimization by addressing it through the concept of limited lookahead control. This research work aims to increase resource providers’ profits by reducing power consumption. Similarly, [28] researched the issue of scheduling for multi-tier web applications related to virtualizing heterogeneous systems to decrease power consumption by maintaining performance. Further, [29] recommended a method on the issue of efficient allocation of power in virtual machines over the complete environment of a virtualized heterogeneous computing system. [30] worked on and used continuous optimization to solve the difficulty of power-aware dynamic placement of applications in interaction with a virtualize heterogeneous environment. [31] have worked on the allocation of available power budgets among servers related to virtualized server farms in heterogeneous environments to decrease the mean response time. Furthermore, they used the proposed model to detect optimal power allocation.

Jung et al. [32] analyzed the issue of dynamic consolidation of virtual machines running on multi-tier web applications while using live migration. However, the proposed method is only implemented on individual web application setups and cannot be used as a service system for multitenant infrastructure. Similarly, [33] worked on the same issue of capacity planning and resource allocation by proposing three controllers: the longest, shorter, and shortest time scales. Every controller operates at various time scales.

Kumar et al. [34] developed a method for dynamic virtual machine consolidation based on estimation stability. Further, they mentioned that the resource demands of application estimation are performed by utilizing the time-varying probability density function. They stated that the values can be achieved by utilizing offline profiling of applications and calibration; however, offline profiling is impractical for infrastructure as a service system. Likewise, [35] researched a similar issue of dynamic consolidation of virtual machine-running applications using machine learning algorithms to optimize the combined energy consumption. However, this method was applied for high-performance computing and is not appropriate for various workloads.

Arshad et al. [36] proposed an algorithm based on energy proficiency heuristics by utilizing virtual machine consolidation to reduce greater usage of energy consumption in the cloud data server environment. They build up a model for virtual machines relocation from one physical host to the other with an aim to lower energy consumption. Moura et al. [37] used the internal value fuzzy logic approach to overcome the problems of resources using vagueness and inaccuracies to save energy with the lowest performance deprivation. They increased energy effectiveness by 2.3% in cloud computing simulation environments. Similarly, Shaw et al. look at the application of reinforcement machine learning to address the virtual machine consolidation issue related to the dissemination of virtual machines throughout the cloud data centers to enhance the management of resources. They enhance energy efficiency by 25% and lower service violations by 63%. Liu et al. [38] proposed a method to overcome the problem of virtual machine consolidation to optimize energy utilization. They presented a new algorithm to choose the optimal solution for energy usage optimization by accomplishing an average conservation of 42% energy. Further, Gharehpasha et al. [39] presented an approach to combine both Sine and Cosine algorithms with the salp swarm algorithm for the best possible virtual machine placement. Also, their research work aims to decrease energy utilization in cloud data centers environment with SLA reduction. Hussain et al. [40] developed a schedule-based algorithm to decrease energy usage in the heterogenous virtual machine cloud environment. After all, Katal et al. [41] conducted a thorough survey on energy efficiency in a cloud computing data center environment. They discussed various methods to lower the power usage in data centers with hardware component level for decreasing the usage by components.

As a variation to the above literature findings, we propose that the central research field consists of single servers and exclusive tasks. Though, presently, huge cloud computing platforms such as Gaikai and Amazon EC2 come up with servers that are spending versatile applications which are further disseminated universally. Conversely, there is an examination disparity in gaming, particularly for multi-player scale games with consumers located remotely. In contrast to this, less evidence has been found regarding energy saving in the context of large data in single-objective applications. The notion of virtualization is employed by researchers using a local regression and robust migration algorithm. The findings propose that latency and service quality can be attained in huge data servers with this virtualization technique. Still, adjustment is a prerequisite between the quality of service and experience [42]. Table 1 shows the comparison among different optimization techniques with an applied method, category, and problem resolution.

## 3. Challenges

The main challenges are explored in two domains such as (1) migration to a single virtual machine and (2) migration to a dynamic virtual machine.

### 3.1. Migration of a Single Virtual Machine

Virtual machines offer benefits to the system consumption, workload, and flexibility of the data center. However, challenges remain, such as waste of resources, network congestion, and consolidation, which will cause server hardware failures. Single virtual machine migration is used by researchers to define a data center with particular properties [43,44]. Similarly, [45] worked to increase the server average utilization and experiments on the historical data to predict the future servers’ demands, as well as migrating the virtual machine in conditions of future needs.

Unstable length and long latency are the key challenges of migrating virtual machines in wide-area networks. Therefore, [46] get significantly responsive in wide area network migration by proposing a three-phase solution. Most importantly, virtual machine migration is widely utilized to conserve power using the consolidation of idle desktop virtual [47]. Moreover, researchers have developed algorithms with the objective of decreasing power mode transition latency [48].

### 3.2. Migration of a Dynamic Virtual Machine

Virtual machine migration (VMM) is the movement of some or all parts of virtual machine data from one place to a different place, with live migration having no interruption of the provided services. VMM is organized in two ways: live migration and non-live migration. In non-live migration, the virtual machine is suspended earlier migration and conditional on whether the virtual machine needs to remain the running services later migration or not. If it is suspended, then the states will be moved into the target site.

**Figure 1:**
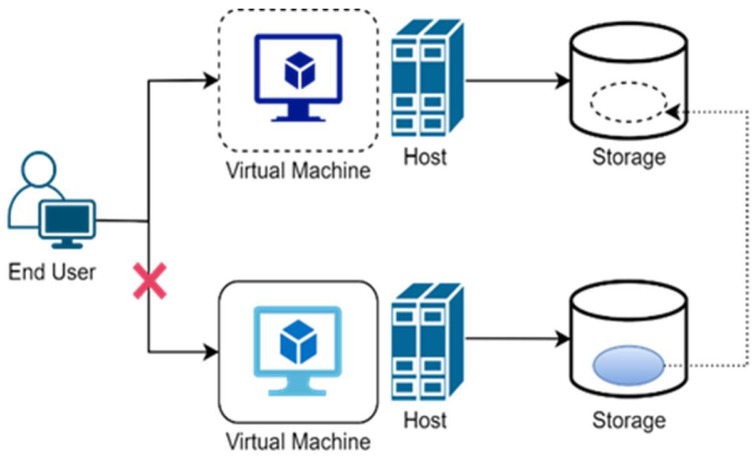
Non-Live Migration.

In the case of migration, all the connections are restored after virtual machine continuation because no open network connection is preserved, as shown in Figure 1.

**Figure 2:**
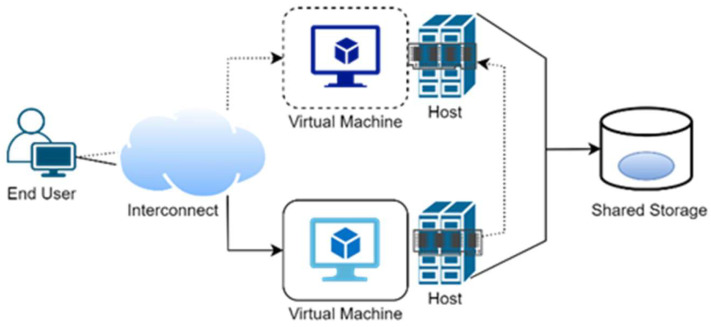
Live VM Migration.

Live migration is the movement of a virtual machine operating on one physical host to a different host devoid of interrupting the usual operations or triggering any stoppage or other undesirable causes for the end user, as shown in Figure 2.

In live migration, data migration memory and network connection continuity are two problems. However, a few challenges are associated with the migration of dynamic virtual machines, such as the consideration of multiple hosts and multiple virtual machines [49]. Other challenges include memory data migration, storage data migration, and network connection connectivity [42].

## 4. System Methodology

**Figure 3:**
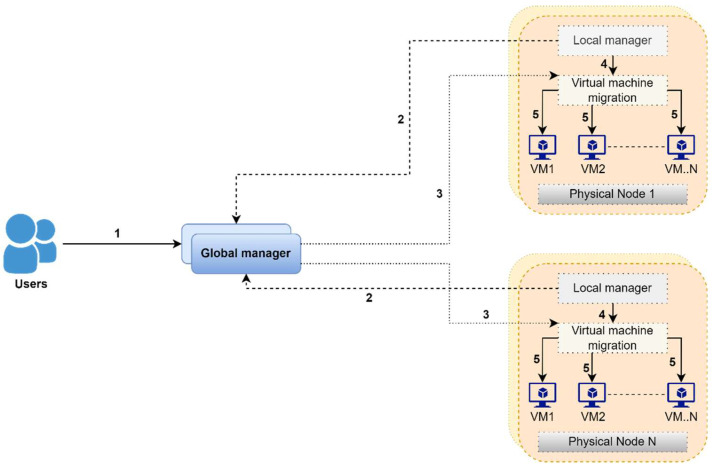
Overall System Methodology. 1 defines the user type as a global user and each node communicates to the global manager through its local manager represented by 2. Each node is divided into the number of VMs represented as 5 that are managed by their local manager for migration presented by 4. The global manager issues commands for the optimisation of the VM assignments shown in 3.

The overall system methodology is shown in Figure 3, which consists of the software layer of the system, which is tied up with local as well as global management modules. Local managers represent individual nodes as a component of the VMM. The main purpose of this is to continuously monitor all the nodes contributing to the CPU utilization and then adjust all resources that are needed for a virtual machine, and finally to decide about the node’s migration timing and place related to a virtual machine, as shown in point 4 of Figure 3.

- The global manager represents a master node to gather information from all local managers to preserve the total layout of the consumption of related resources, as shown in point 2 of Figure 3.
- The global manager provided instructions for the optimization of virtual machine positioning, as shown in point 3 of Figure 3.
- The main function of VMMs is to resize and migrate the virtual machines and shift the power modes of the nodes, as presented in point 5 of Figure 3.

## 5. Performance Analysis and Discussion

Some tests have been conducted on CloudSim simulation settings to determine different characterizations of resource optimization. All these tests were executed on the same datasets by applying “Eclipse Luna and Java IDE Developers. 283 MB; 144,793 DOWNLOADS”. Different optimization techniques have been used, namely dynamic voltage and frequency techniques, non-power awareness, and static virtualization techniques. These tests have been designed and carried out on a data set from World of WarCraft that is a massively multiplayer online games (MMOs) game that is multi-location multi-environment. Test environments consist of multiple avatars over 3.5 years collected from an online cloud environment. This helps to test the limits of resource optimization for cloud environments for different features, such as energy optimization. service level agreement, service level agreement violations, virtualization, host timing, etc. Virtualization techniques will be used for the management of load for virtual machines (VMs) that are over or underloaded in the system, and relocation of these will be performed based on techniques such as minimum migration time (MMT), maximum correlation (MC), and minimum utilization (MU).

DVFS, non-power aware (NPA), and static threshold virtualization technique (STVM) techniques will be compared in the same environment. For STVM techniques, defined resources are used in terms of random-access memory (RAM), bandwidth, storage, and input-output file size, whereas in dynamic technique, resources are allocated based on central processing unit voltage and frequency fluctuations.

Different evaluation metrics will be used to gauge the performance of the proposed system. Initially, the tests are divided into different techniques for example DVFS, NPA, and STVM. The reason for dividing them into sub-techniques is to see how the proposed system will behave under different conditions. Test environment and workload are standard for all methods. All these proposed methods will be measured against certain defined parameters such as energy consumption, VM selection time, VM relocation time, host selection meantime, and service level agreement violations. These matrices will help to determine which technique will perform better under static and dynamic workloads in the proposed test environment. The comparison method will also help to determine which technique performs better for energy saving and resource optimization for small and large servers placed globally.

**Figure 4:**
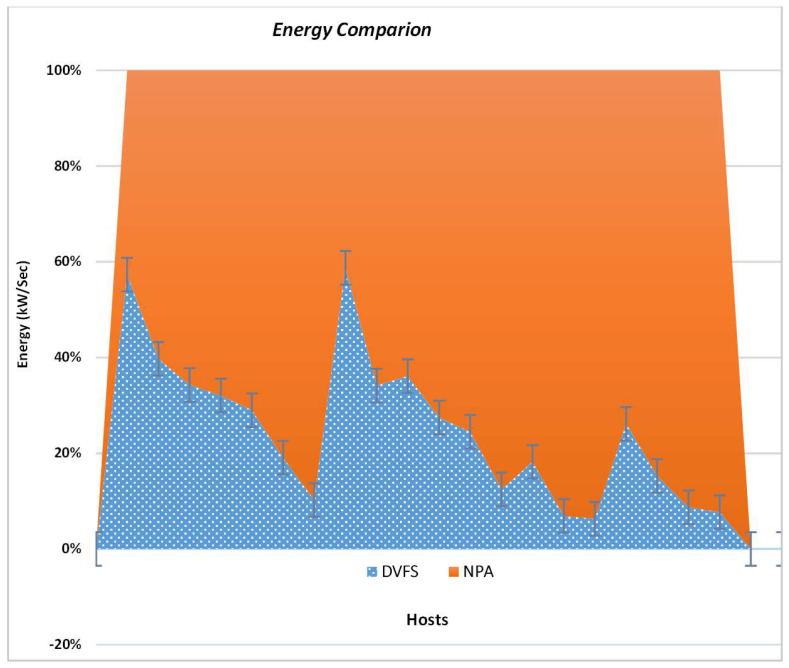
Illustrations of Energy Utilization in a Data Center.

A test has been carried out to distinguish how dynamic frequency scaling will behave with non-power-aware techniques for the same workload. The results in Figure 4 are plotted using the reality check method. The results show that the non-power-aware method consumes more power compared to the dynamic voltage and frequency methods. DVFS shows a linear trend for energy consumption and less consumption of power. The DVFS method results in increased profits and minimum SLAs per host compared to the NPA technique. However, using NPA with the same host numbers and fixed millions of instructions per second (MIPS) consumes more energy in the setup, emitting higher CO_2_ emissions.

**Figure 5:**
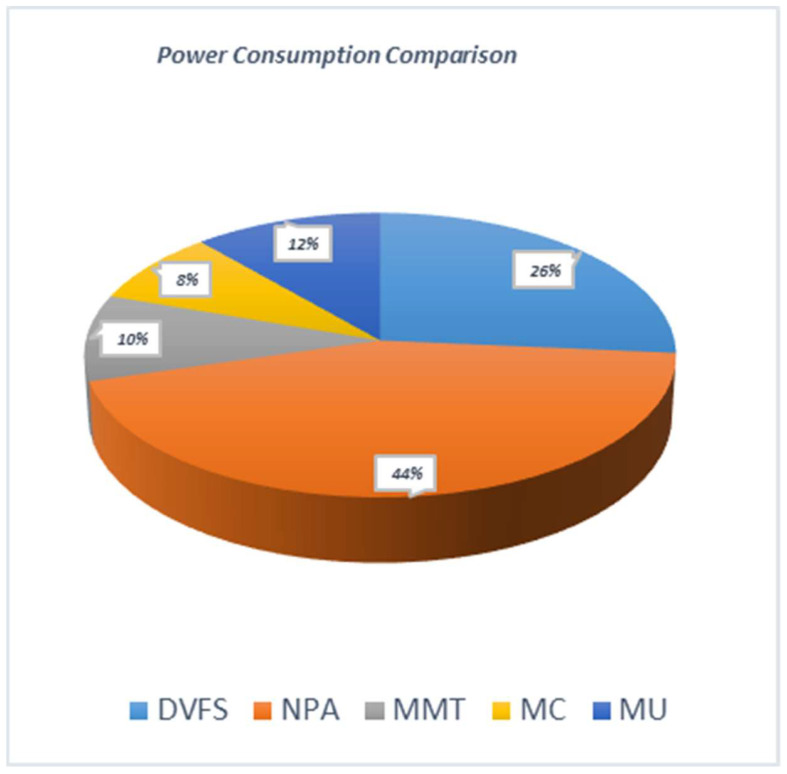
Evaluation of Energy Utilization in the Recommended System.

A similar test is further extended, and the static threshold virtualization technique (STVM) has been added to determine the energy consumption. In these experimental results, as shown in Figure 5, three virtualization techniques were used to relocate the virtual machines for overloaded and underloaded hosts. This relocation of virtual machines is done using minimum migration time (MMT), minimum correlation (MC), and maximum utilization (MU) in a static threshold environment.

In STVM, higher, and lesser threshold boundaries are specified for any test environment. In the static threshold technique, MC has less energy consumption compared to the MU or MMT method. When compared with the dynamic voltage and frequency techniques, the results are different. Static threshold behaves better for small workloads as upper and lower limits are definable for required parameters. In comparison to the dynamic workload environment, DVFS again proves to have less service level agreement violation (SLAV) and maintains higher SLAs, resulting in a better quality of service and better user experience compared to the NPA method. It can also be concluded that STVM virtual machine relocation methods are supported with smaller workloads, which verifies the theoretical concept.

**Figure 6:**
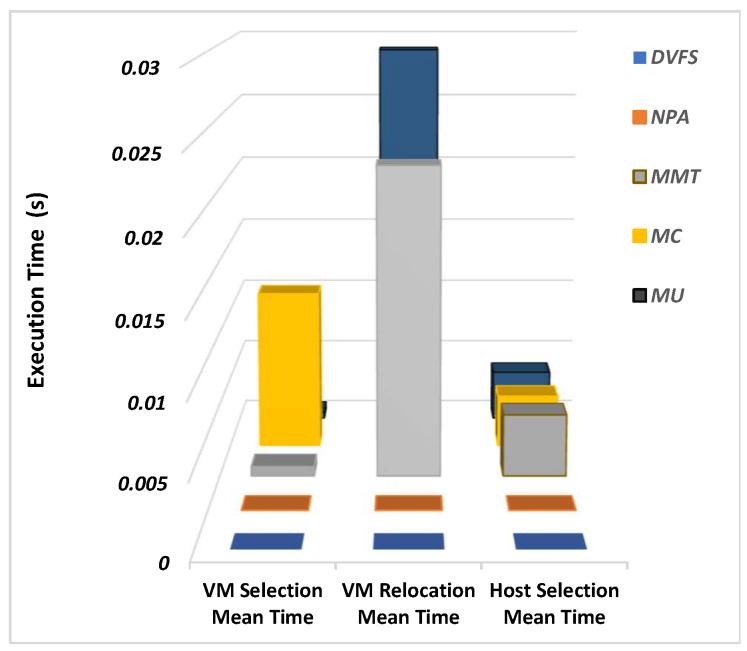
Virtual Machine Performance Time for Every Host.

All three techniques are used to compare the execution times for three techniques for different levels of hosts with the same configuration setup in Figure 6. Virtual machine selection, relocation, and host selection time remained similar for DVFS and NPA.

MC has the highest VM selection time in a static environment, and MC takes more time for VM relocation when compared with other techniques. In a static environment, all three techniques have similar host selection meantime because of defined threshold limits as compared to a dynamic environment. The results also support the theoretical concept that no relocation of VMs is done for DVFS, and resource optimization is done using central processing unit (CPU) voltage and frequency methods.

**Figure 7:**
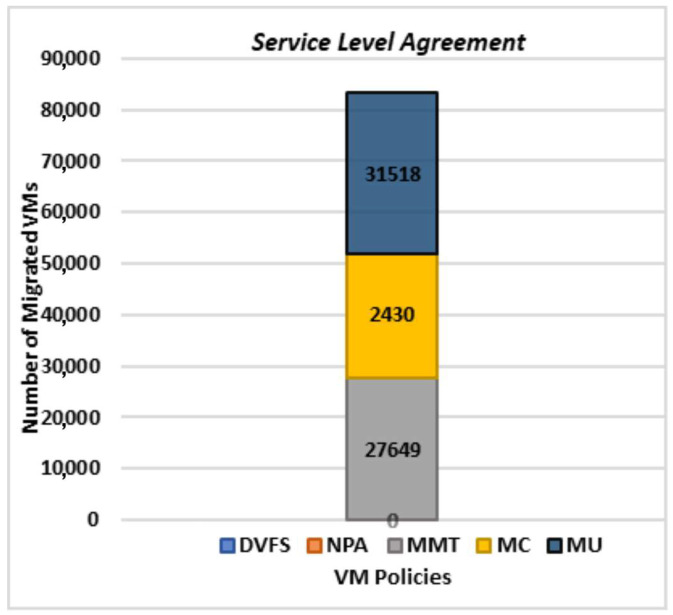
Sum of Virtual Machine Migrations.

If a proper virtualization technique is selected, downtime in the network can be reduced for overloaded and underloaded environments. The results in Figure 7 show that in the STVM method, MC has the lowest number of virtual machines that are migrated, whereas maximum utilization has the highest number of migrations. NPA and DVFS do not carry any VM migrations, which second the theoretical concept of dynamic voltage and frequency scaling and non-power aware techniques.

**Figure 8:**
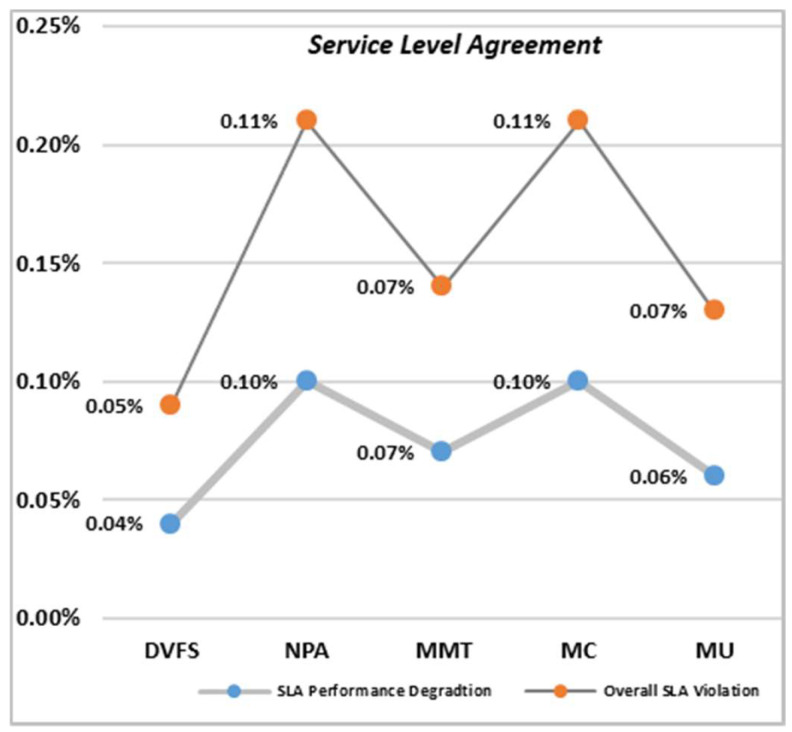
Analysis of the Service Level Agreement Violation.

Service level agreement and service level agreement degradation were administered for all three techniques. DVFS has a minimum service level degradation when compared to the rest of the techniques. NPA has the highest number of SLAV. If better service quality is required, fewer SLAV methods need to be selected. The MMT technique needs to be selected for a better user experience, as this has a minimum number of SLAVs and SLAs for the static threshold environment, as shown in Figure 8.

In dynamic environments, DVFS has less energy consumption associated with NPA methods. In a static environment, MMT has the highest number of host shutdowns, as VMs are selected and relocated for loaded hosts to save resources and energy. MMT, therefore, also has less mean and standard deviation time in a static environment compared to other virtual machine relocation techniques.

**Figure 9:**
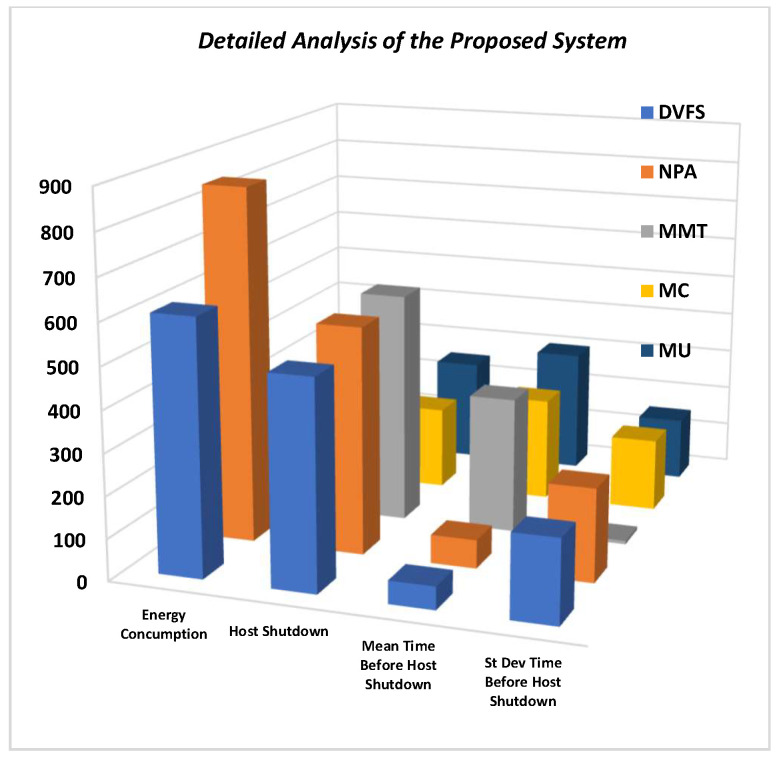
Overall detailed evaluation of the developed system.

Therefore, the overall detailed analysis of the proposed system is shown in Figure 9. So, depending on whether the test environment is dynamic or static, resource optimization, service quality, and better user experience can be achieved if proper methods are selected for loaded hosts in a cloud environment. Proper selection of optimization techniques will help in energy and resource optimization for large-scale servers that are placed and operating globally.

## 6. Conclusions

Different simulation experiments are designed using the CloudSim simulation environment to test resource optimization in cloud gaming servers. These experiments suggest different resource optimization techniques for large and small servers. Gaming datasets are versatile in nature and consist of different audio, video, avatars, locations, etc. The data versatility helps to challenge resource optimization in terms of energy consumption, execution time, virtual machine relocation, and service level agreement violations for different user levels.

From the results, it is evident that different resource optimization techniques are required to be selected for under-and overloaded hosts depending on servers and user data type. If the data that is being processed has defined limits, then the static threshold technique will be used with another virtualization discussed above. In terms of a dynamic environment with multiple users and a large pool of resources, dynamic resource optimization behaves better. Therefore, for large servers, DVFS saves more energy, has fewer service level agreement violations, and results in a better quality of service and experience.

In the future, this work will be enhanced to explore new energy-saving techniques and compared them with the current methods. This work will also be extended to other domains of computing for example Internet of Things (IoT), Big Data, and Artificial Intelligence (AI).

## Figures and Tables

**Table 1:** Different Optimization Techniques.

Method	Categories	Technique	Resolves
Data Centre Resource Management [26,27]	Local and Global Policies	Virtualization	Sequential optimization by addressing it through the concept of limited lookahead control
Scheduling for multi-tier web applications [28]	Virtualizing heterogeneous systems	Virtualization	Decreases power consumption by maintaining performance for multi-web applications
Power-aware dynamic placement of applications [30]	Dynamic Virtualization	Continuous Optimization	Power-aware dynamic placement of applications in interaction with a virtualized heterogeneous environment
Dynamic virtual machine consolidation [34]	Dynamic VM consolidation based on estimation stability	Resource demands by utilizing the time-varying probability density function	Resolves resource optimization for small applications
Dynamic Voltage and Frequency (DVFS)—Proposed	Single and Multi-server	DVFS, based on workload	Saves power and resolves resource optimization issues based on workload for servers placed locally and globally

## Data Availability

Data will be available upon request through correspondence email.
